# A case for using spatial evidence in waterborne disease control

**DOI:** 10.2471/BLT.26.296233

**Published:** 2026-06-01

**Authors:** Zhuo Li, Hezhishi Jiang, Paul AN Ngwakum, Kun Zhai, Minghui Ren

**Affiliations:** a Institute of Area Studies, Peking University, Beijing, China.; b United Nations Children’s Fund Eastern and Southern Africa Regional Office, Nairobi, Kenya.; c Department of Global Health, School of Public Health, Peking University, No.38 Xueyuan Road, Haidian District, Beijing, 100191, China.

Cholera affects approximately 47 low- and middle-income countries, with up to 4 million cases and 143 000 deaths annually.^1^ Spatial risk maps now exist for most high-burden settings, yet this geographic knowledge has not been matched by equivalent progress in converting spatial evidence into implementation decisions. A structural gap persists between what surveillance systems reveal and how interventions are designed, sited and adjusted. Importantly, surveillance must be complemented by prevention measures to balance the diagnostic focus with proactive strategies that reduce transmission risk before outbreaks occur.

Modelling across sub-Saharan Africa demonstrated that roughly 4% of districts account for the majority of the region’s cholera cases, and that targeting interventions in these areas could eliminate half the regional burden.^2^ The Global Task Force on Cholera Control codified this geographic distribution into its 2030 roadmap, centring strategy on priority areas for multisectoral interventions.^3^

The epidemiological rationale for geographic targeting is well established, but frameworks for translating spatial knowledge into decisions are underdeveloped. Hotspot identification does not indicate how to configure a treatment network across a dense informal settlement, which communities require mobile delivery rather than fixed facilities or how to redistribute resources as an outbreak moves along a transmission corridor. Crucially, hotspot identification should also feed into preparedness frameworks. 

Spatial epidemiology has contributed to understanding the geographic distribution of waterborne disease risk through high-resolution mapping and environmental modelling.^2^^,^^4^ Yet spatial health research remains predominantly diagnostic and does not address how interventions should be designed differently for different settings. Implementation science has established context as a primary determinant of intervention success,^5^ but context within these frameworks does not necessarily incorporate the structured variation that geography introduces. Moreover, spatial data alone cannot ensure uptake unless communities are actively engaged in planning and trust-building; without this social dimension, even well-targeted interventions risk limited effectiveness.

We propose a spatial lens as a bridge between the fields of spatial epidemiology and implementation science. A spatial lens recognizes the geographic conditions of the intervention location and shapes whether, how and for whom an intervention works. Importantly, this lens should also integrate climate risk projections such as floods and droughts, which are critical determinants of cholera outbreaks. In this way, interventions can be better aligned with predictable environmental stressors that drive transmission.^4^

Assessments in the African Region suggest that progress in translating spatial intelligence into coordinated water, sanitation and hygiene infrastructure investment remains slow.^6^ Cholera control inherently requires action across multiple sectors: health systems, water and sanitation authorities, education ministries and municipal planning.^7^ A spatial lens offers the common operational picture that can align these sectors around shared geographic priorities. Preparedness measures can further ensure that geographic intelligence translates into timely and anticipatory action. For example, in United Republic of Tanzania, the disaster response coordination unit sits in the Prime Minister’s Office to bring together health and water ministries, and the government developed a multisectoral approach focusing on 17 high-risk geographic areas.^8^

Four domains illustrate where a spatial lens adds decision-relevant value. First, facility siting. Treatment centres and water, sanitation and hygiene infrastructure are frequently located based on administrative convenience rather than systematic assessment of population coverage. Location-allocation methods can identify configurations that maximize reach. Prevention-oriented domains are equally critical, ensuring that interventions address root causes of transmission rather than only treatment needs. Second, spatiotemporal constraints. Road conditions deteriorate during rainy seasons precisely when cholera transmission risk peaks, creating predictable periods of reduced service reachability.^4^ Third, locally differentiated strategies. Dense urban settlements, peri-urban areas and rural communities present distinct combinations of transmission dynamics and infrastructure availability, requiring calibrated intervention mixes.^9^ Fourth, dynamic outbreak response. Cholera transmission follows spatial pathways shaped by water systems and population movement; therefore, integrating real-time case data with geographic information enables more responsive resource deployment.^10^

Research is needed on how to establish effective multisectoral spatial decision-sharing platforms that enable hotspot identification to trigger coordinated responses across sectors.^11^ Yet sustaining such coordination requires robust financing and governance mechanisms; without these, spatial intelligence risks remaining underutilized and fragmented. Any infectious disease shaped by the geographic conditions of water, sanitation and human settlement will benefit from implementation frameworks that take geography as a determinant of what is possible, equitable and effective.
